# FABP5-binding lipids regulate autophagy in differentiated SH-SY5Y cells

**DOI:** 10.1371/journal.pone.0300168

**Published:** 2024-06-20

**Authors:** Alejandro Soto-Avellaneda, Alexandra E. Oxford, Fabio Halla, Peyton Vasquez, Emily Oe, Anton D. Pugel, Alyssa M. Schoenfeld, Matthew C. Tillman, André Cuevas, Eric A. Ortlund, Brad E. Morrison

**Affiliations:** 1 Biomolecular Sciences Ph.D. Program, Boise State University, Boise, ID, United States of America; 2 Department of Biological Sciences, Boise State University, Boise, ID, United States of America; 3 Department of Biochemistry, Emory University, Atlanta, GA, United States of America; Xiangtan University, CHINA

## Abstract

The motor features of Parkinson’s disease result from loss of dopaminergic neurons in the substantia nigra with autophagy dysfunction being closely linked to this disease. While a large body of work focusing on protein effectors of autophagy has been reported, regulation of autophagy by lipids has garnered far less attention. Therefore, we sought to identify endogenous lipid molecules that act as signaling mediators of autophagy in differentiated SH-SY5Y cells, a commonly used dopaminergic neuron-like cell model. In order to accomplish this goal, we assessed the role of a fatty acid-binding protein (FABP) family member on autophagy due to its function as an intracellular lipid chaperone. We focused specifically upon FABP5 due to its heightened expression in dopaminergic neurons within the substantia nigra and SH-SY5Y cells. Here, we report that knockdown of FABP5 resulted in suppression of autophagy in differentiated SH-SY5Y cells suggesting the possibility of an autophagic role for an interacting lipid. A lipidomic screen of FABP5-interacting lipids uncovered hits that include 5-oxo-eicosatetraenoic acid (5OE) and its precursor metabolite, arachidonic acid (AA). Additionally, other long-chain fatty acids were found to bind FABP5, such as stearic acid (SA), hydroxystearic acid (HSA), and palmitic acid (PA). The addition of 5OE, SA, and HSA but not AA or PA, led to potent inhibition of autophagy in SH-SY5Y cells. To identify potential molecular mechanisms for autophagy inhibition by these lipids, RNA-Seq was performed which revealed both shared and divergent signaling pathways between the lipid-treated groups. These findings suggest a role for these lipids in modulating autophagy through diverse signaling pathways and could represent novel therapeutic targets for Parkinson’s disease.

## Introduction

Parkinson’s disease (PD) is the second most frequent neurodegenerative disease and is characterized by motor system dysfunction caused by loss of dopaminergic (DA) neurons in the substantia nigra. Macroautophagy (subsequently denoted as autophagy) plays an important role in PD as evidenced by disease-causing mutations in genes directly involved in this essential cellular process [1]. Autophagy is a fundamental process for degrading macromolecules and organelles and is also the only cellular process known to remove large protein aggregates [2]. Therefore, defective autophagy is believed to be associated with the pathophysiology of PD due to the presence of hallmark Lewy body inclusions [3]. As a result, autophagy has been of a keen focus for the development of PD therapy.

Autophagy is a highly ordered catabolic process that begins by targeting material to be degraded through conjugation of p62 or by other mechanisms [4]. Once targeted, the material is engulfed by a lipid vesicle derived from the endoplasmic reticulum. The lipid vesicle is created through the propagation of the phosphatidylethanolamine-Atg8 (LC3) lipoprotein complex. A vesicle with fully enveloped cargo will fuse with multivesicular bodies and then lysosomes possessing digestive enzymes to degrade the cargo into simple constituents that can be recycled for metabolic purposes. This process occurs constitutively within cells; however, the rate and targeted cargo can be modulated by numerous signaling pathways, many of which converge on a master regulatory complex known as TORC1 [5].

Advances in our understanding of autophagy have occurred largely through the investigation of protein effectors of this process. However, little is known about the contribution of endogenous lipid compounds to autophagy regulation. Principal knowledge in this field has arisen from the study of AKT pathway activation by phosphatidylinositol phosphorylation, largely in the context of growth factor signaling and oncogenesis. Despite the reduced focus in this area, the involvement of phosphatidylinositol indicates that lipids are indeed critical signaling mediators for autophagy control. A limited understanding of other lipid contributors is likely due to the inherent physical properties of these biomolecules, which include enhanced oxidation, degradation, and insolubility, rendering them challenging to study. Despite these limitations, lipid research is becoming more accessible, largely as a result of advancements in mass spectrometry resources (e.g., methods, databases) and the commercial availability of lipids. As such, we have employed these tools to identify autophagy-relevant lipids.

There are 10 members of the FABP family (FABP1-9 and 12) present in humans. This protein family represents a strong pool of potential bait proteins for the identification of autophagy-regulating lipids because these members transport fatty acids and other lipophilic compounds (e.g., eicosanoids and retinoids within cells) [6, 7]. Furthermore, in *C*. *elegans*, an FABP homolog binds to lipids derived from autophagy and regulates lifespan and autophagic processes [8]. Individually, these proteins have both distinct and shared cargo lipids. The unique functionality of specific FABP members is also suggested by diverse tissue and developmental expression as well as associations with human disease [6, 9].

A detailed examination of individual members has led to our specific interest in FABP5. Ewing et al., have provided evidence that FABP5 interacts with a known autophagy-regulating protein (ATG5) as one of 368 bait proteins in a mass spectrometry-based interactome screen [10]. In addition, FABP5 expression has been reported to modulate PI3K/AKT signaling [9]. This is of interest since PI3K/AKT signaling plays a central role in regulating autophagy via mTOR activity. Furthermore, FABP5 might have particular relevance to PD since it has been reported that its expression is highly enriched in nigral DA neurons [11]. As a result, we began our investigation into autophagy-regulating lipids by focusing on FABP5 interactors.

## Materials and methods

### Cell culture

SH-SY5Y human neuroblastoma cells, from ATCC, were cultured in T175 tissue culture flasks and kept at 37°C with a 5% CO_2_ environment. Cells were cultured in DMEM/F12 50/50 mix (Corning Cellgro) with L-glutamine, 15% Fetal Bovine serum (Atlanta Biologicals), 1% non-essential amino acids (Corning Cellgro), 1% Penicillin-Streptomycin solution (HycloneTM, 1000 units/mL Penicillin- 10000μg/mL Streptomycin) as previously described by Shipley, Mangold, & Szpara [12]. Cells were passaged when the flasks reached ~80%-90% confluency using 0.25% Trypsin, with 2.2mM EDTA and sodium bicarbonate (Corning). Cells at passage number 5–15 were used for experiments. For experiments, cells were plated on multi-well tissue culture plates at the necessary concentrations (e.g., 3 million cells per well on a 6-well plate). Before experimentation, the cells were treated with 10 μM retinoic acid for 7 days, to differentiate them into dopaminergic neuron-like cells. HEK 293T Human Embryonic Kidney cells were grown in DMEM media with 1.0 g/L glucose with L-glutamine and sodium pyruvate (VWR), 10% Fetal Bovine Serum (Atlanta Biologicals), and 1% Penicillin-Streptomycin solution (HycloneTM, 1000 units/mL Penicillin- 10000μg/mL Streptomycin) and passaged as described for SH-SY5Y cells.

### Immunohistochemistry

All mouse work performed in this research was approved by the Boise State University IACUC (approval # AC20-005). Mice to be processed for immunohistochemistry were be deeply anesthetized with isoflurane by nose cone. Transcardiac perfusion was performed with a 25 gauge needle inserted into the left ventricle with lacerated right atrium. Infusion of 40mL of phosphate buffer (PB) with 1mM EDTA occurred followed by 40mL of 4% paraformaldehyde in PB and tissues were then harvested. Tissues were then dehydrated in 30% sucrose/PB at 4°C for 3 days, dabbed dry with Chemwipes, quickly frozen in OCT media on dry ice and then stored at -80°C. Tissue was then sectioned to 20 μm onto Superfrost glass slides using a Leica cryostat. Sections were dried in a drawer for 1 hour and then processed for immunohistochemistry. Immunohistochemistry was performed as described previously [13]. Briefly, slides containing sections were permeabilized, blocked with antibody solution (5% Donkey serum in Tris-Buffered Saline with 0.05% Tween 20 (TBST)), probed with FABP5 antibody (Cell Signaling Technology # 39926) and Tyrosine Hydroxylase antibody (Cell Signaling Technology # 45648) overnight at room temp (RT), washed 3 times with TBST, probed with anti-rabbit secondary antibody conjugated to Alexa 594 (Thermo Fisher, A32754) or with anti-mouse secondary antibody conjugated to Alexa 488 (Thermo Fisher, A21202) for 2 hours at RT, washed 2 times with TBST, then once with TBS, then incubated for 10 min at RT with Hoechst (1:10000), then washed once with PB and coverslips mounted with EverBrite Antifade medium (Biotium). Sections were then viewed using an using an EVOS M5000 fluorescence imaging system.

### Lipid treatment of cells

Stock solutions of 5-oxo-ETE (10mM), hydroxystearic acid (100mM), arachidonic acid (100mM), palmitic acid (100mM), and stearic acid (100mM) were made by dissolving each lipid in sterile 95% ethanol and stored at -80°C. Stock and dosage concentrations were chosen by using similar concentrations as those used in other published studies within the literature [14–16]. During experimentation, SH-SY5Y cells were treated with each relevant lipid or the vehicle control (ethanol) dissolved into a media solution consisting of DMEM/F12 50/50 mix (Corning Cellgro) with L-glutamine, 15% freshly thawed Fetal Bovine serum (Atlanta Biologicals), 1% Penicillin-Streptomycin solution (HycloneTM, 1000 units/mL Penicillin- 10000 μg/mL Streptomycin) for 4 hours followed by lysis.

### Detergent-free immunoprecipitation

FABP5-V5 tagged, overexpressing SH-SY5Y cells were plated onto a 100 mm cell culture dish and treated with retinoic acid for 7 days. Cells were then lysed using a freeze-thaw lysis technique. Before lysis, cells were washed twice with phosphate buffer. Then cells were frozen at -80°C for 15 minutes in 120 μL PBS. Cells were then thawed and scraped into microcentrifuge tubes. Following lysis, the cells were centrifuged at 15000 g for 10 minutes at 4°C and the supernatant was collected. The supernatant was incubated with either V5 antibody (D3H8Q, Cell Signaling Technology, 13202S) or IgG isotype control antibody (Cell Signaling Technology, 3900) for 1 hour at 4°C on rotation. Following primary antibody incubation, ChIP grade protein G magnetic beads (Cell Signaling Technology, 9006S) were added and the supernatant was rotated at 4°C. Then the magnetic bead complexes were pelleted and washed twice with PBS and once with ddH2O. Water was then removed, and magnetic bead pellets were resuspended in a 2:1 chloroform: methanol solution and sent for lipidomic analysis at The Emory University Integrated Metabolomics and Lipidomics Core Facility.

### Targeted mass spectrometry analysis of lipid interactors

Samples were thawed and resuspended in 200 μl methanol and vortexed at 300 rpm for 15 minutes. The clean methanol extract was transferred to the clean glass vial after centrifuging the methanol extract at 4000 rpm for 10 minutes. The samples were reextracted twice with 300 μl methyl tert-butyl ether and 300 μl MTBE: MeOH (3:1 v/v) and dried under a gentle stream of nitrogen. The combined lipid extract was then treated with 250 μl of oxalyl chloride, incubated at 65°C for 5 minutes, and dried under nitrogen stream. Further, the dried residue was treated with 1% 3-picolylamine, incubated at room temperature for 5 minutes, and dried under nitrogen stream. Finally, the derivatized samples were resolved in 200 μl methanol prior to analysis.

The mass spectral data were acquired by injecting 5 μl in triplicate into a SCIEX QTRAP 5500 mass spectrometer via an Exion LC AC autosampler. A precursor ion scan of m/z 109.1 was performed to target all the carboxylic acid-containing lipids followed by additional MS/MS fragmentation to enhance lipid identification. A total of eight features were observed and annotated after subtraction of background signals relative to blank injections. The eight features include fatty acids, hydroxy fatty acid, and an inflammatory lipid. One of the features (m/z 772) was not identified and the feature at m/z 409 is putatively identified as either HEPE or 5OE.

### Protein expression and purification

Full-length human FABP5 in the pMCSG7 vector was transformed into *Escherichia coli* strain BL21(DE3) cells and expressed as a His_6_ fusion containing a tobacco etch virus protease cleavage site to facilitate tag removal [17]. Cultures (1 liter in TB) were grown to an *A*_600_ of ∼0.8 and induced with 0.5 mm isopropyl β-d-1-thiogalactopyranoside at 18°C for ~18 hours. Cell mass was harvested, lysed through sonication in a buffer containing 20 mM Tris HCl pH 7.4, 150 mM NaCl, 25 mM imidazole, 5% glycerol, lysozyme, Dnase A, and 100 uM phenylmethylsulfonyl fluoride, and purified by nickel affinity chromatography. The His-tag was cleaved by tobacco etch virus protease at 4°C overnight with simultaneous dialysis into a buffer containing 20 mM Tris HCl pH 7.4, 150 mM NaCl, and 5% glycerol and purified to homogeneity by nickel affinity followed by gel filtration chromatography using a HiLoad 16/60 Superdex 75 column into 137 mM NaCl, 2.7 mM KCl, 10 mM Na_2_HPO_4_, 2 mM KH_2_PO_4_ (pH 8.0).

### *In-vitro* fluorescent binding assay

Ligand binding to FABP5 was measured via competition of 1-anilinonaphthalene-8-sulfonic acid (1,8-ANS), a small molecule whose fluorescence increases drastically when surrounded by a hydrophobic environment and has been shown to bind an array of FABPs with varying affinity [17]. Briefly, binding of 1,8-ANS was carried out in PBS (137 mM NaCl, 2.7 mM KCl, 10 mM Na_2_HPO_4_, 2 mM KH_2_PO_4_, pH = 8.0) in the presence of 250 nM 1,8-ANS with increasing protein concentrations ranging from 10 nM to 400 μM. Blank measurements obtained from protein-only samples were subtracted at each protein concentration tested to obtain the final values and the resulting fluorescent values were fit with a One-Site binding curve to determine the binding constant, K_D_. Competition assays with 12-hydroxystearic acid and 5-Oxo-ETE were then performed in the same buffer system in which the protein was held at a constant concentration of 500 nM, and 1,8-ANS also being held constant at 10 μM in the presence of increasing fatty acid concentrations from 12 nM to 400 μM via 50x ethanol stocks to maintain an ethanol concentration of 2%. Blanks consisting of 1,8-ANS and fatty acid in the absence of protein were subtracted at each ligand concentration tested. The resulting fluorescence values were used to calculate a *K*_*i*_ value for the fatty acid of interest. Following a 30-minute incubation at 37°C, data were collected on a BioTek Synergy NEO plate reader using an excitation wavelength of 380 nm and an emission wavelength of 480 nm and processed in GraphPad Prism 7. All curves are the average of three independent replicates.

### Western blotting

Before western blotting, cells were washed with PBS, lysed with RIPA lysis buffer containing a protease inhibitor cocktail, and subsequently sonicated at 80% intensity for 7 seconds. Following sonication, cells were centrifuged at 15000g for 10 minutes at 4°C, and the supernatant was then collected. Polyacrylamide gel electrophoresis was then performed on the 15 μg of protein extracts on either a 4–12% or 16% pre-cast gel, depending on the size of the protein assayed and the band separation required for proper analysis. Electrophoresed proteins were transferred onto a PVDF membrane using the iBlot2 Dry transfer device and transfer materials. The transfer was performed according to the manufacturer’s instructions with the iBlot2 device. Original western blot images and raw data can be accessed at Open Science Framework (https://osf.io/e7am4).

Primary antibodies were used to detect LC3 (Novus Biologicals, NB100-2220), FABP5 (Cell Signaling Technology, 39926S), and β-actin (Thermo Fisher, MA1-91399). Anti-rabbit (Cell Signaling Technology, 7074) and anti-mouse HRP secondary antibodies (Cell Signaling Technology, 7076) were used to probe western blot PVDF membranes after primary antibody incubation. Pierce ECL Western Blotting substrate (Thermo Scientific) was then used to develop membranes. Membranes were imaged on a Bio-Rad blot imager. Protein expression was analyzed by taking densitometry measurements on the images obtained, using ImageJ and Microsoft Excel.

### Lentiviral production and transduction

Thermo Fisher Virapower kit was used to produce lentivirus. HEK 293T Human embryonic Kidney cells (ATCC) were transfected with the transgene-carrying plasmids (custom from VectorBuilder, Inc. or Addgene), together with plasmids containing lentiviral packaging components (VSVg, LP1, and LP2), using lipofectamine 2000 (Life Technologies). Lentiviral packaging plasmids LP1 and LP2, as well as coat protein plasmid VSV and the desired transgene plasmid vector (7.2 μg of each), were incubated with Lipofectamine 2000 in Opti-MEM media (Gibco) for 20 minutes at room temperature. The mixture was then added dropwise to flasks containing 293T cells in fresh media. The next day, media was replaced with fresh media. The following day, the supernatant containing full assembled, active lentivirus was collected and filtered through 400nm pore filters. Filtered supernatant media was aliquoted as needed and stored at -80°C until ready for use. For transduction into target cells, aliquoted virus-containing media was added directly to the media bathing the target cells in their flasks. The following day, media was replaced with fresh media. Puromycin-based selection for shRNA constructs was initiated three days after viral addition and maintained for the duration of the experiment.

### LC3B-GFP-mCherry puncta assessment

SH-SY5Y cells were plated into 24-well plates containing poly D-lysine coated coverslips. Cells were transduced with lentiviral stocks created using the FUW mCherry-GFP-LC3 plasmid (Addgene, plasmid # 110060) [18]. The following day virus-containing media was replaced with fresh media. 48 hours after initial transduction cells were differentiated into neurons with 10 μM RA for 7 days. Following the differentiation period, cells were treated with relevant lipids for 4 hours. Treated cells were then fixed with 4% paraformaldehyde (in PBS) for 23 minutes on ice. The glass coverslips with attached cells were then washed with PBS and incubated in 1:10000 Hoechst dye in PBS for 5 minutes on ice and washed again in the dark. Finally, the coverslips were mounted on glass slides for fluorescent imaging using Fluoromount G. Glass slides were imaged on a Leica Stellaris 5 Confocal with the automated z-stack function. Red only puncta and green only puncta were counted with ImageJ on each image produced and a value for flux was determined as the ratio of red only puncta to total puncta for each image. This processing was automated via Image J to create objective quantification of puncta.

### RNA extraction

SH-SY5Y cells were plated onto 6-well plates and treated with either vehicle control (ethanol), 5OE (40 μM), SA (400 μM), or HSA (400 μM) for 6 hours. Following treatment, cells were lysed using the materials included in the RNeasy RNA extraction Minikit (Qiagen, 74104). Total RNA was extracted with included materials according to the manufacturer’s protocol. RNA concentrations and purity were measured using a spectrophotometer (Implen Pearl nanophotometer).

### RNA Seq

Extracted RNA was sent to Novogene for sequencing and analysis. Briefly, RNA was poly-A captured (rRNA depleted), RNA fragmented and cDNA synthesized. Sequencing was performed using an Illumina NovaSeq 6000 system. Analysis was performed for paired-end 150 bp reads with ≥ 20 million reads per sample. A differential gene expression analysis was performed by Novogene using the DESeq2 R package and KEGG and Reactome annotated pathways enrichment was determined. Sequence reads for this experiment have been deposited in the SRA under BioProject accession PRJNA768785.

## Results

### FABP5 is expressed by dopaminergic neurons in the substantia nigra

**Fig 1:**
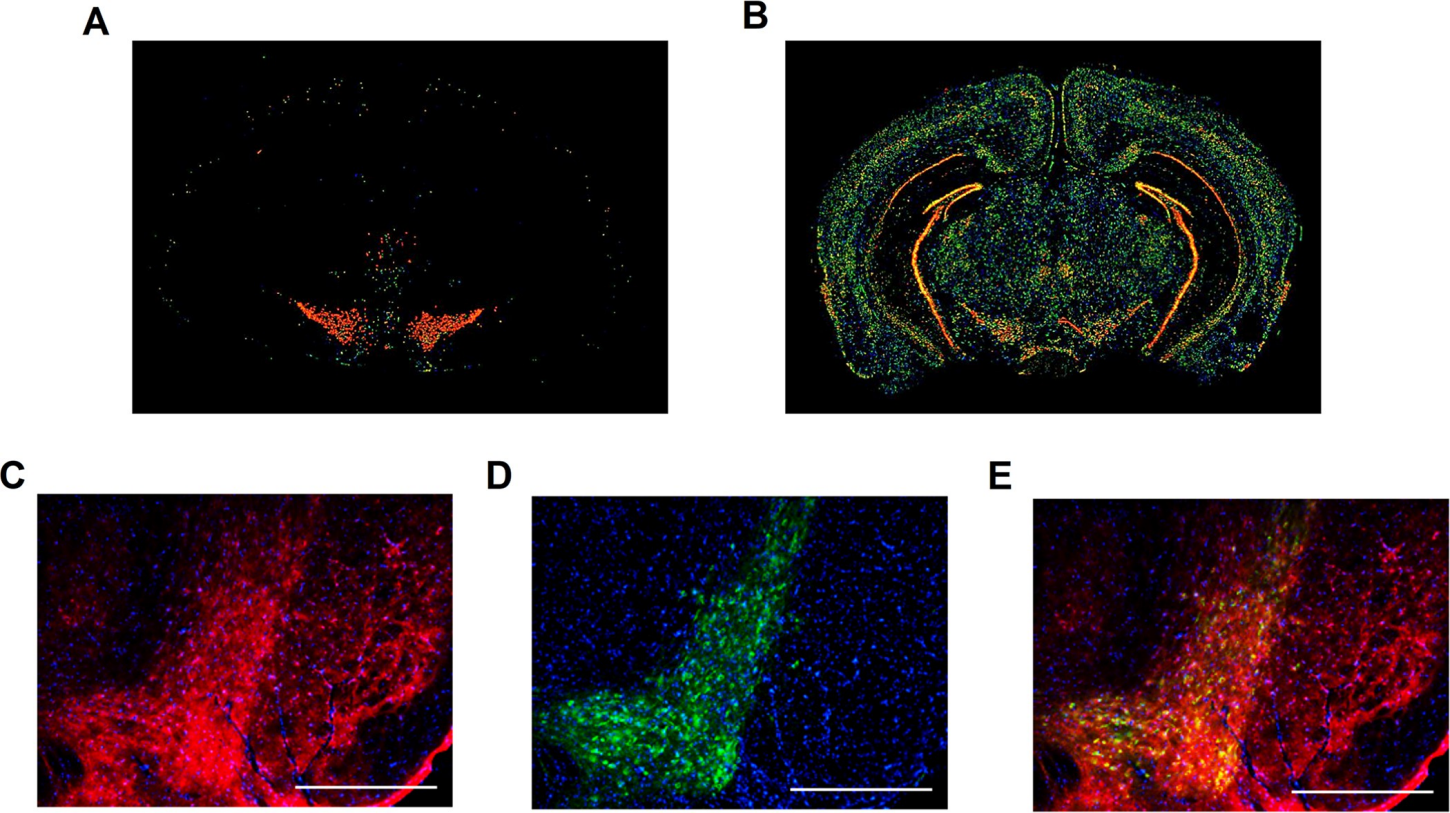
FABP5 is expressed by nigral dopaminergic neurons. *In situ* hybridization for *Tyrosine hydroxylase* **(A)** and *Fabp5* **(B)** mRNA expression [Mouse Brain Atlas, Allen Brain Institute]. Immunohistochemistry was performed for FABP5 **(C)** and Tyrosine Hydroxylase **(D)** in adult mouse brain substantia nigra with nuclei labeled using DAPI and merged image shown **(E)**. Scale bar = 400 μm.

We utilized *in situ* hybridization data, provided by the Allen Brain Institute to help select the appropriate FABP member for our study. Of the FABP members examined by The Allen Brain Institute, FABP5 was the only transcript to exhibit robust expression in the substantia nigra **(Fig 1A and 1B)** [19]. We then confirmed FABP5 protein expression by dopaminergic neurons in the substantia nigra using immunohistochemical analysis of adult mouse midbrains **(Fig 1C–1E)**. For these reasons, we chose to examine in FABP5 using SH-SY5Y cells, a human neuroblastoma cell line that can be differentiated into post-mitotic dopaminergic neuron-like cells [20, 21].

### FABP5 regulates autophagy

**Fig 2:**
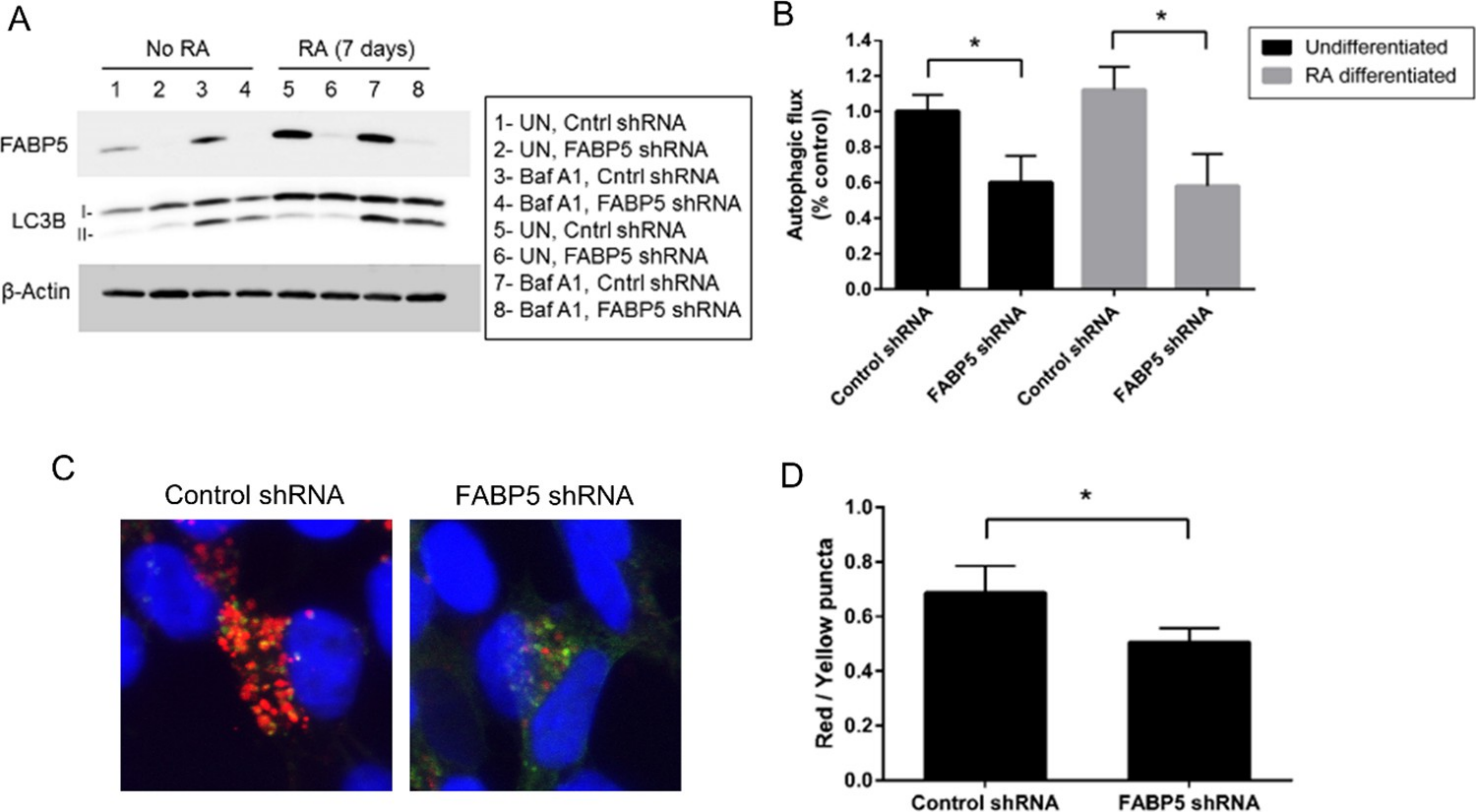
FABP5 regulates autophagy. **(A)** Undifferentiated or RA-differentiated SH-SY5Y cells were transduced with lentivirus containing scrambled (Cntrl) or FABP5 shRNA constructs and LC3B-II expression evaluated by western blot and **(B)** autophagic flux determined by densitometry of the relative abundance of LC3B-II/β-Actin. **(C)** Representative images for mCherry-GFP-LC3B that was transduced by lentivirus into RA-differentiated control or FABP5 knockdown as described in **A** is shown and then autophagosome (yellow) and lysosomes (red) quantified. **(D)** Graphical depiction of red and yellow puncta is presented. 20 cell images were taken per condition per biological replicate (6 biological replicates were performed). *p<0.05, error bars = SEM.

**Fig 3:**
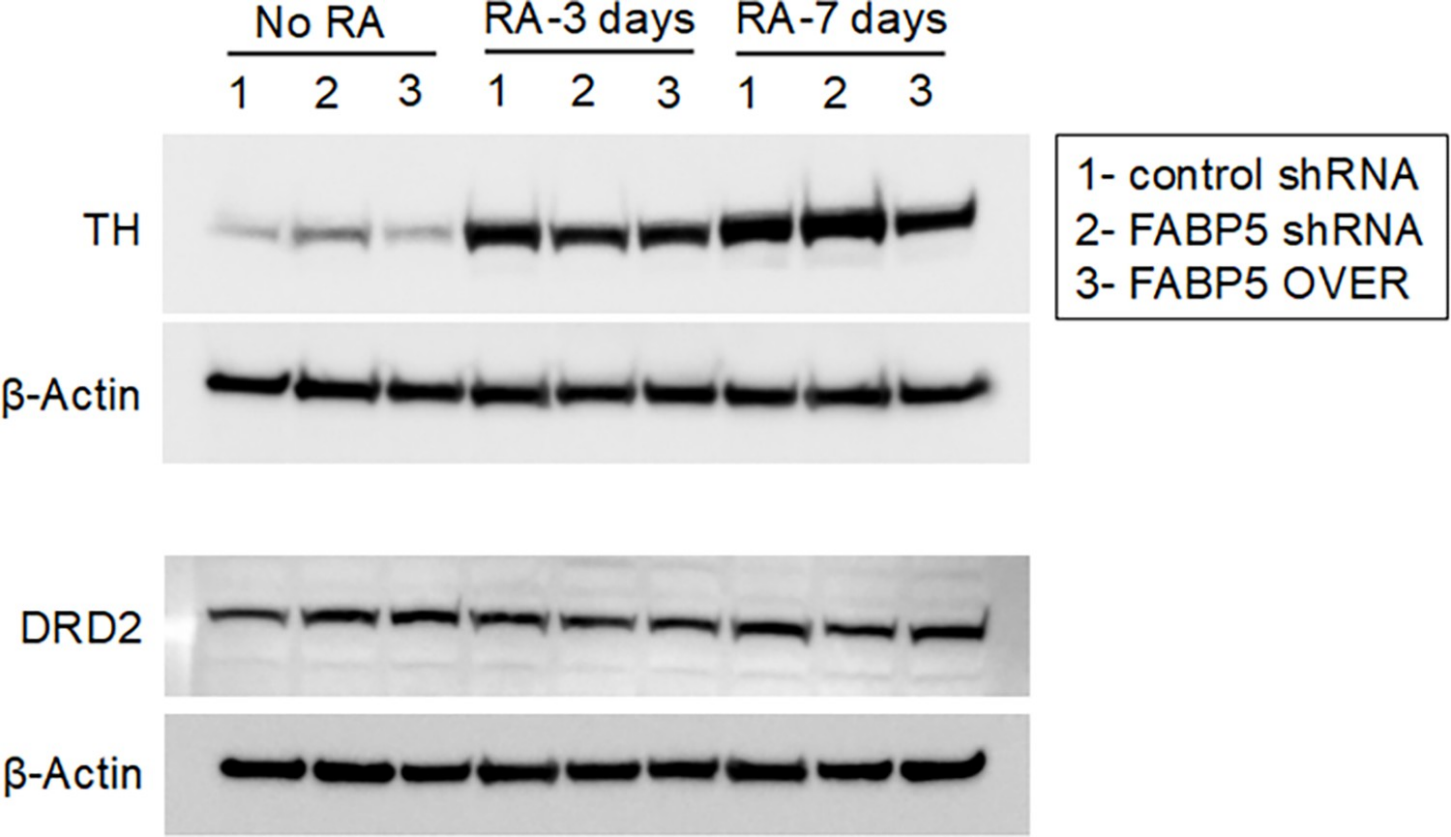
FABP5 does not affect SH-SY5Y differentiation by RA. SH-SY5Y cells were transduced with lentivirus containing a scrambled (control) shRNA, FABP5 shRNA or an FABP5 overexpression (OVER) construct. Cells were selected for transgene expression using puromycin and treated with all-trans RA (10 μM) for the indicated times and Tyrosine Hydroxylase (TH) and Dopamine receptor D_2_ (DRD2) protein levels were assessed by western blot. The figure shows 3 biological replicates (1, 2, 3).

To address whether FABP5 affects autophagy, we modulated the expression of FABP5 and assessed its effect on autophagic flux. To that end, we engineered SH-SY5Y cells to stably express scrambled or FABP5 shRNA. A population of transgenic cells was then obtained by selection with puromycin. SH-SY5Y cells were then differentiated into post-mitotic dopaminergic neuron-like cells with 7 days of 10 μM of all-trans retinoic acid (RA) supplemented to growth media as previously described [12, 22, 23]. Autophagic flux was determined by comparing the autophagosome marker LC3B-II (normalized to β-Actin) in untreated versus bafilomycin A1 treated cultures **(Fig 2A and 2B)** [24]. We observed a decreased abundance of LC3B-II in the bafilomycin A1 treated cultures when FABP5 expression was knocked down indicating reduced flux. We also monitored autophagic flux in a secondary assay by quantifying autophagosome/lysosome ratios observed in mCherry-GFP-LC3B transgene-expressing cells **(Fig 2C and 2D)** as previously described [24, 25]. We observed a reduced autophagosome/lysosome ratio in mCherry-GFP-LC3B expressing cells when FABP5 is knocked down. These findings were corroborated using a second, distinct shRNA targeting FABP5 **(S1 Fig)**. Taken together, this data indicates that FABP5 knockdown results in repressed autophagy. In addition, we found that neither FABP5 knockdown nor overexpression affected SH-SY5Y differentiation **(Fig 3)**, indicating that differences in these cells were not due to altered RA signaling or differentiation status.

### Long-chain fatty acids interact with FABP5

**Fig 4:**
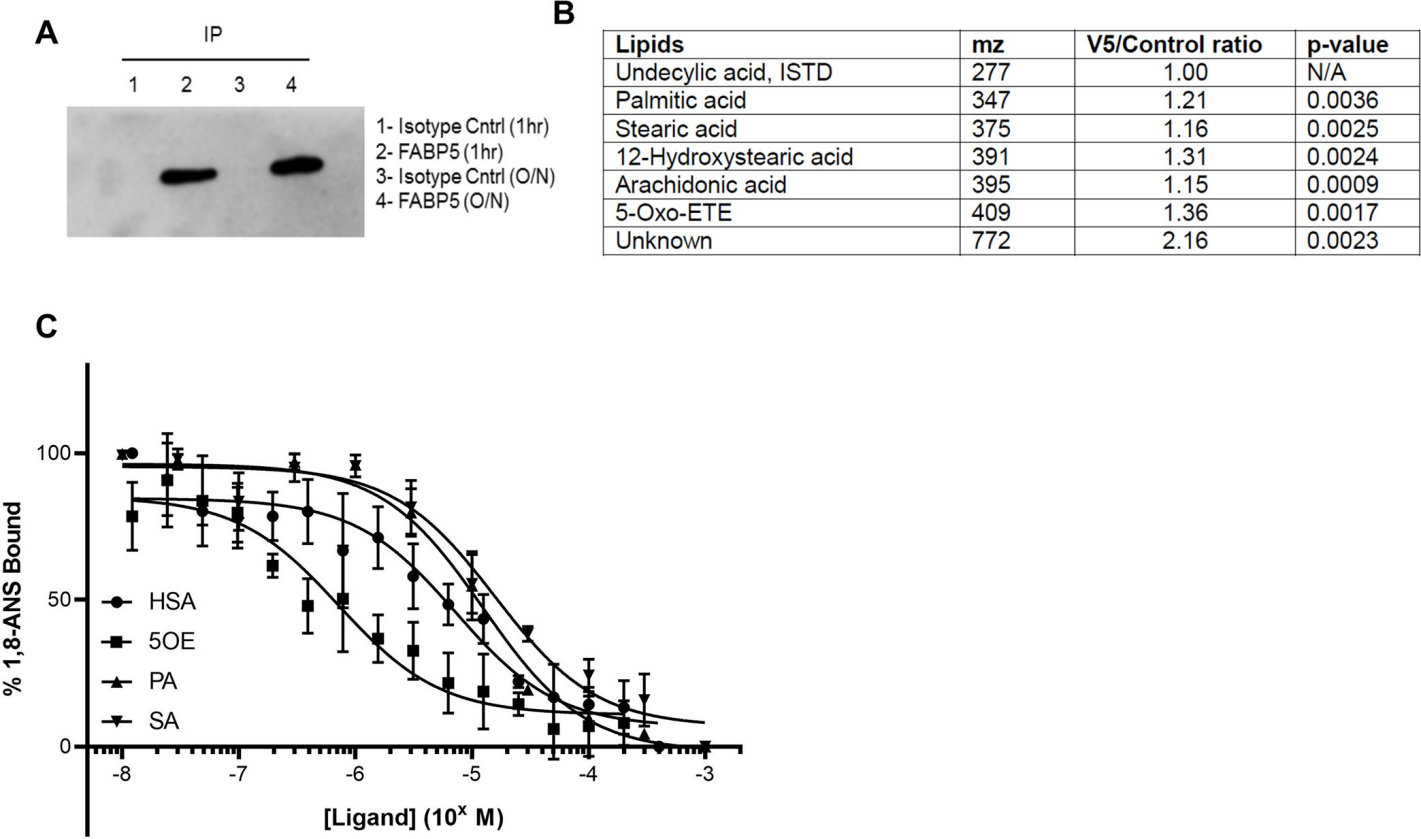
FABP5 lipidomic interactome. **(A)** FABP5 was successfully immunoprecipitated (IP) with a detergent-free method using lysate obtained from RA-differentiated SH-SY5Y cells that stably express a recombinant FABP5-V5. Immunoprecipitations (V5 epitope tag-specific or antibody isotype control) were performed for 1 hr or overnight (O/N) and western blotting was performed using an FABP5 antibody. **(B)** Samples obtained by 1 hr FABP5-V5 IP (V5) or control antibody IP (Control) from 3 independent biological replicates were processed for lipid extraction and analyzed by QTRAP 5500 mass spectrometer via Exion LC AC autosampler. Six lipids were identified to have significant interaction with FABP5. **(C)** Fluorescent hydrophobic reporter 1,8-anilinonapthalenesulfonate (ANS) was preincubated with recombinant FABP5. Increasing doses of HSA, 5OE, PA and SA were added and bound ANS measured by fluorescence.

Having established an autophagy-regulating function for FABP5, we next employed FABP5 as a bait protein to identify lipid interactors. We created an SH-SY5Y cell line using a lentivirus that stably expressed FABP5 with a V5 epitope tag. A detergent-free immunoprecipitation protocol was developed to co-purify FABP5 and associated lipids **(Fig 4A)**. Cells were RA-differentiated for 7 days and then treated with media containing fresh 15% FBS for 1 hour. FABP5-lipid complexes were then purified and sent to the Emory University lipidomics core for analysis. Six lipids (one unidentifiable by mass) were enriched in the V5-FABP5 cells relative to the IgG isotype controls **(Fig 4B)**. Arachidonic acid (AA) was identified in this group and is well-studied as a precursor for a diverse set of metabolites, including 5-Oxo-eicosatetraenoic acid (5OE), another FABP5 interactor found in this analysis. Other long-chain fatty acids were also identified that including hydroxystearic acid (HSA), stearic acid (SA), and palmitic acid (PA).

Both AA and PA were previously reported to specifically bind to FABP5 [17], however, 5OE, HSA, and SA were novel interactors. In order to confirm the interaction between these lipids and FABP5, we performed an *in vitro* binding assay. The fluorescent hydrophobic reporter anilinonapthalenesulfonate was used with recombinantly expressed and purified FABP5. Increasing doses of 5OE, HSA, PA and SA were added and the fluorescence of ANS bound to FABP5 was measured. Competitive binding was observed for all 4 lipids, with 5OE binding with ten times higher affinity (Ki = 0.387 μM) than HSA (Ki = 4.32 μM) followed by PA and SA (**Fig 4C).**

### Autophagy inhibition by 5OE, HSA, and SA but not AA or PA

**Fig 5:**
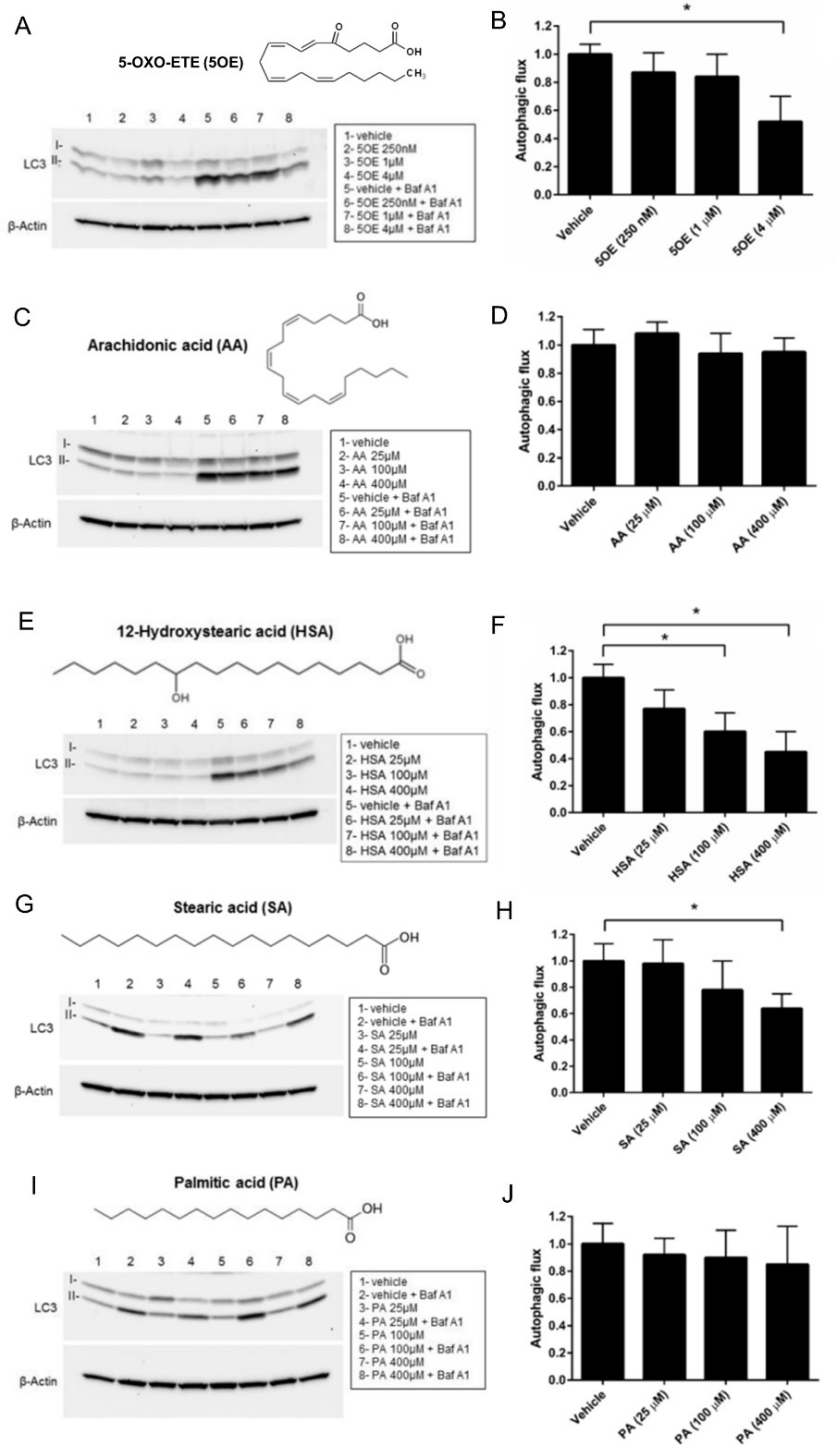
Effect of candidate lipids on LC3B-II autophagic flux. **(A,C,E,G,I)** Increasing amounts of lipid or vehicle (ethanol) were added to RA-differentiated SH-SY5Y cells for 4h in the presence or absence of bafilomycin A1 (Baf A1) and western blotting was performed and autophagic flux was determined **(B, D, F, H, J)** from 3 biological replicates. *p< 0.05, error bars = SEM.

**Fig 6:**
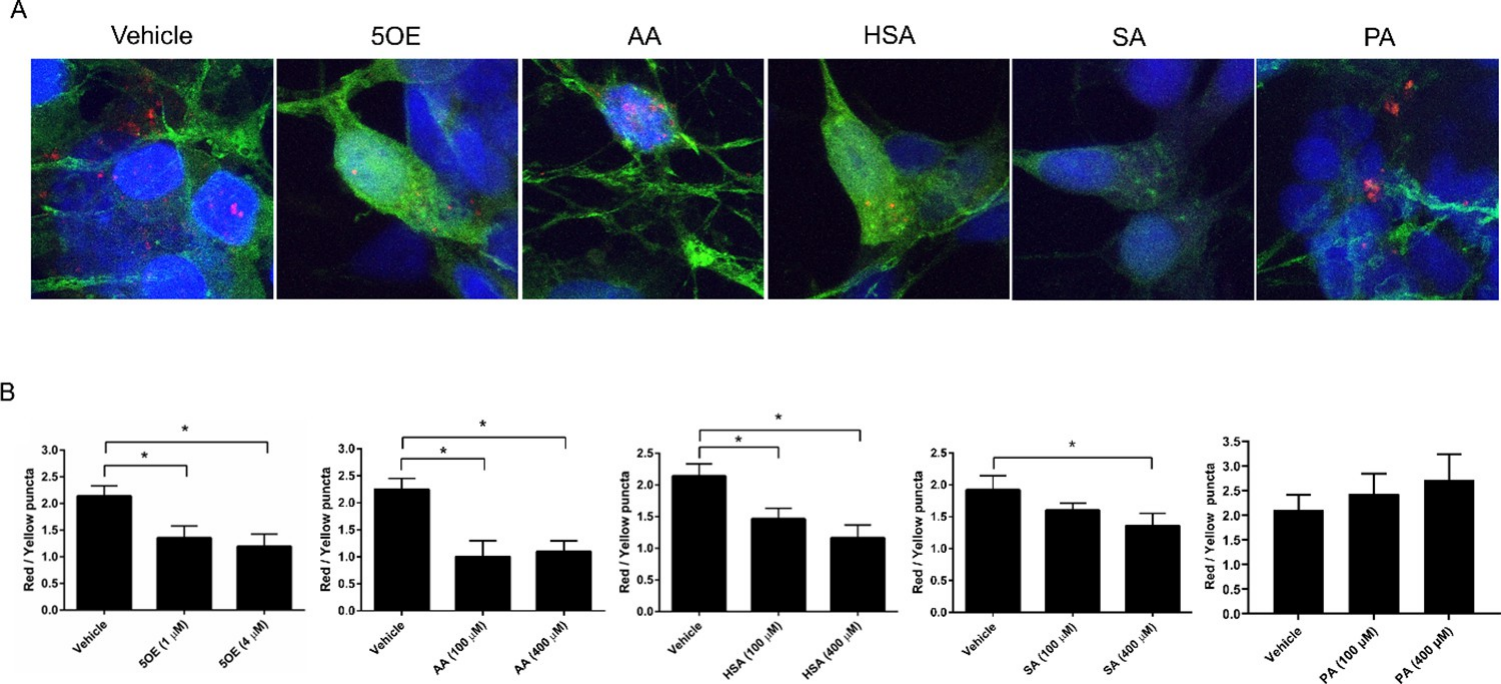
Effect of candidate lipids on LC3B puncta autophagic flux. Lentivirus was used to transduce SH-SY5Y cells with mCherry-GFP-LC3B followed by RA treatment. Seven days later, cells were treated with ethanol (vehicle) or lipid at the highest two doses for 4h. Representative images **(A)** are shown for the highest lipid dose and autophagosomes (yellow) and lysosomes (red). **(B)** Red and yellow puncta were quantified using 20 cell images taken per condition per biological replicate (6 biological replicates in total). *p < 0.05, error bars = SEM.

We next examined whether each of these five candidates affected autophagy in differentiated SH-SY5Y cells. We found that 5OE, HSA, and SA, but not AA or PA, exhibited potent dose-dependent anti-autophagic activity for both LC3-II accumulation and mCherry-GFP-LC3B puncta assays at concentrations reported in human serum as well as in other systems **(Figs 5 and 6)** [14–16]. The concentrations that seemed to elicit the most robust response were 400 μM for HSA and SA and 4 μM for 5OE. This is consistent with the observation that 5OE has a higher affinity for FABP5 than HSA, SA and PA **(Fig 4C).** Interestingly, AA shows an altered lysosome/autophagosome ratio following quantification of mCherry-GFP-LC3B puncta **(Fig 6)** indicative of autophagy inhibition. However, an examination of LC3-II levels following bafilomycin treatment **(Fig 5C and 5D)** reveals no effect on autophagic flux. We observed that at 4 hours of treatment large numbers of cells were detached in the two higher doses of AA, suggesting toxicity. Given many shared mechanisms between autophagy and apoptosis, these results suggest AA induces death rather than autophagy [26].

### OE, HSA, and SA do not induce cell death

**Fig 7:**
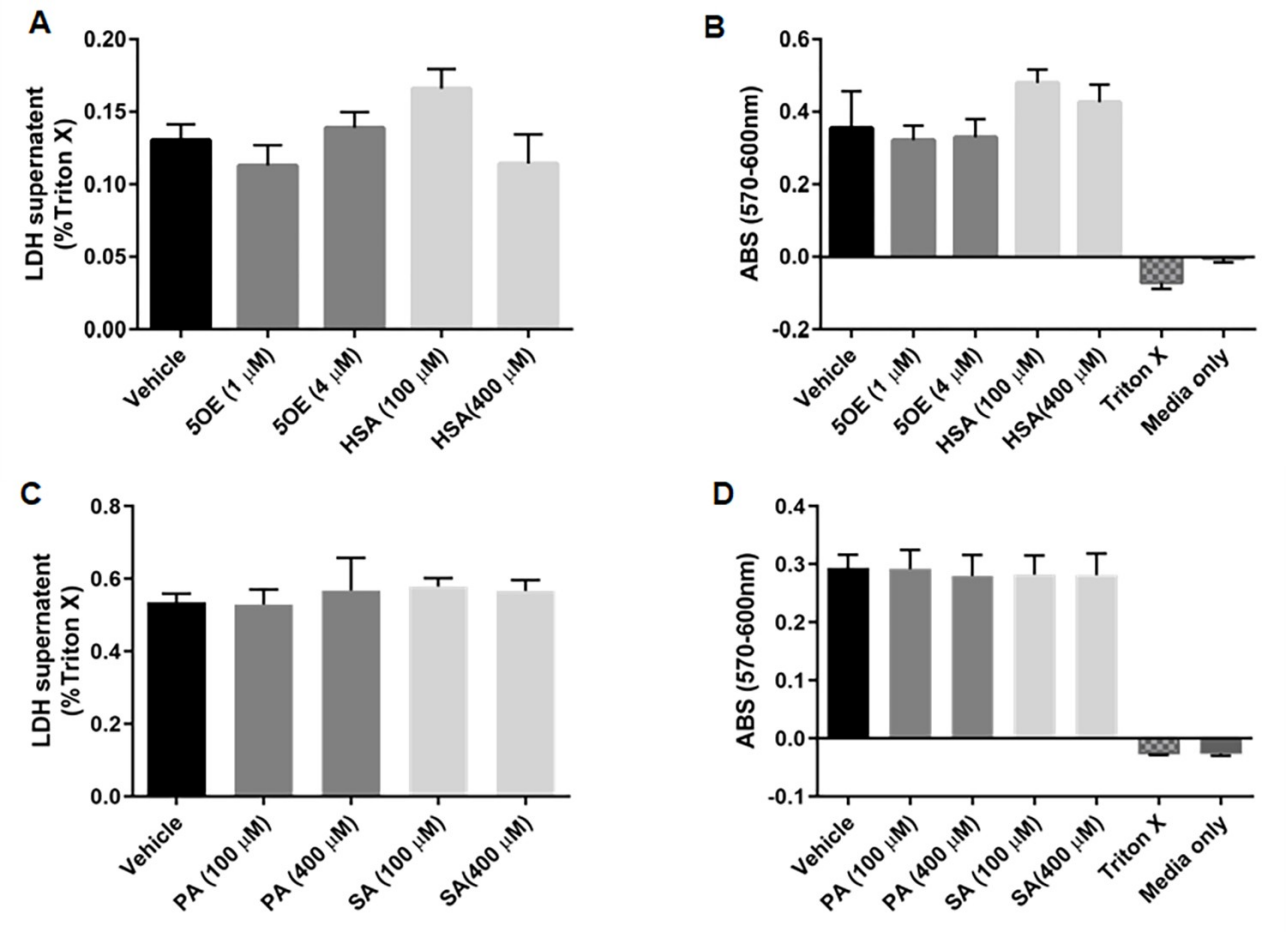
5OE, HSA, SA, and PA do not induce cell death. RA-differentiated SH-SY5Y cells were treated with ethanol (vehicle), 5OE, HSA, SA, or PA as shown in normal growth media. After 24h, cell death was measured by **(A, C)** LDH released into the culture media as well as by **(B, D)** resazurin assay absorbance (ABS). Three biological replicates per condition were performed. *p < 0.05, error bars = SEM.

Autophagic mechanisms can share features with apoptosis [26]. Therefore, we examined whether the lipids, 5OE, HSA, or SA, which exhibited an autophagy-regulating profile in both autophagic flux assays **(Figs 5 and 6)** induced death in differentiated SH-SY5Y cells. These lipids did not exhibit toxicity after 24 hours of treatment **(Fig 7)** indicating that apoptosis is not confounding the autophagic flux data. Interestingly, despite structural similarity, AA showed robust toxicity at 100 μM and 400 μM doses suggesting divergent effects with 5OE.

### Transcriptomic analysis following FABP5-binding lipid treatment

**Fig 8:**
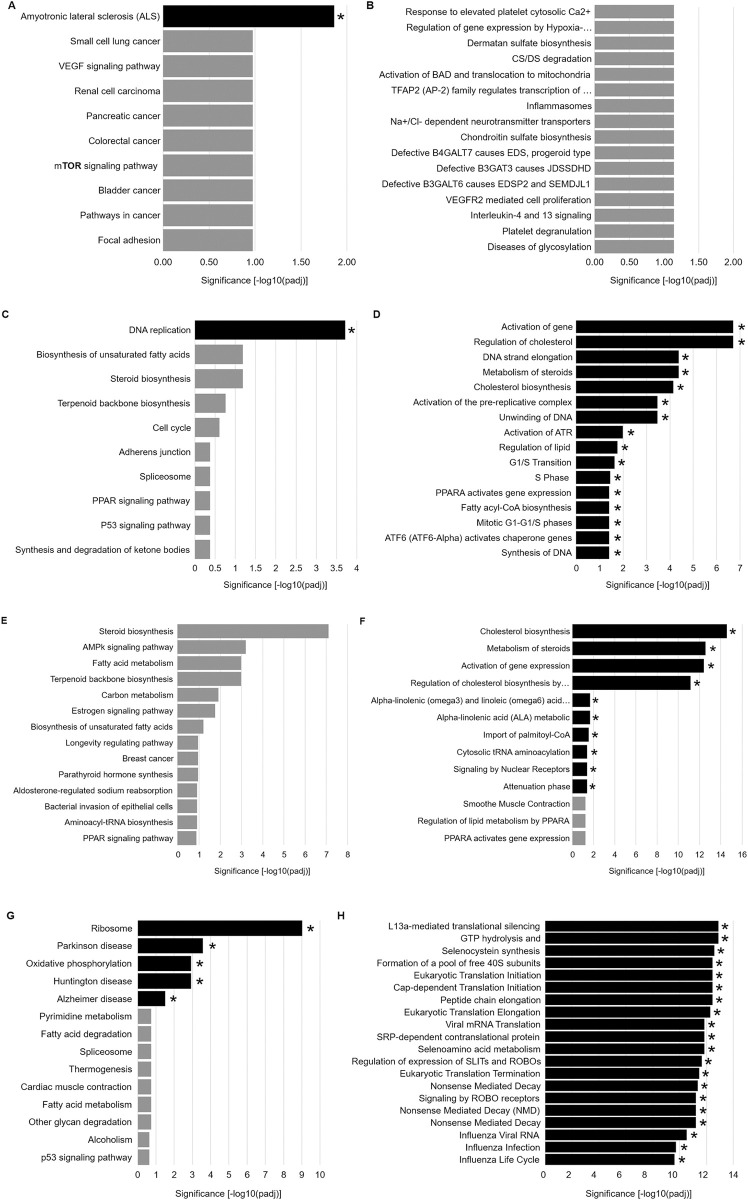
RNA Seq analysis of lipid treatment. KEGG annotated pathways are shown for RA-differentiated SH-SY5Y cells treated with **(A)** 4 μM 5OE, **(C)** 400 μM HSA, **(E)** 400 μM SA, and **(G)** 400μM PA for 6h. **(B, D, F, H)** Reactome pathway enrichment is also shown on the right for each lipid treatment. *Significance versus vehicle treatment group; 3 biological replicates.

We next sought to gain insight into the molecular mechanism of action for autophagy-inhibiting lipid candidates. The short time frame (4 hours) of lipid treatment and autophagy assessment indicates that gene transcription is not the driver of autophagy inhibition within this window. However, affected signaling pathways could leave transcriptomic clues to their identity. Therefore, RNA Seq (random primed with 44M-60M reads/sample) was performed on RA-differentiated SH-SY5Y cells treated with vehicle (ethanol) or lipid (5OE: 4 μM, HSA: 400 μM, SA: 400 μM, and PA: 400 μM) for 6 hours. The 6-hour time point was chosen to better capture transcriptomic changes that might be induced by affected pathways in the 4-hour autophagy inhibition window. Unbiased analysis for transcript enrichment for KEGG and Reactome annotated pathways was performed **(Fig 8).** Substantial numbers of differential transcripts for both HSA and SA-treated cells were identified **(S2 Fig)**. However, only a very modest number of differential transcripts in the 5OE group was found **(S2 Fig).** This lower number of differential transcripts reduces the confidence and pathway associations (amyotrophic lateral sclerosis) from the 5OE data set. For this reason, we also listed the top associated pathways that were not significant. Interestingly, the autophagy-regulating mTOR signaling pathway appeared as a leading but non-significant hit. Both HSA and SA exhibited associations with cell cycle regulation, fatty acid metabolism, and peroxisome proliferator-activated receptor (PPAR) signaling. FABP5 is known to signal via PPAR pathways which are known to regulate autophagy response, thus this might represent a shared mechanism for autophagy inhibition by HSA and SA. Additionally, PA was comparatively examined as a long chain fatty acid that did not exhibit autophagy regulation. Interestingly, PA-treated cells exhibited enriched genes involved in Parkinson’s disease, Alzheimer’s disease, Huntington’s disease associated pathways. However, there were no links to autophagy-associated pathways which is consistent with the lack of autophagic effect observed by PA in this study.

## Discussion

Despite intense efforts to harness autophagy for therapeutic benefit, very few clinical applications have been realized [27, 28]. Consequently, the strong association between autophagic dysfunction and neurodegenerative diseases like PD has spurred investigation into more diverse targets for autophagy regulation [29]. This rationale prompted us to devise a lipidomic screen for the identification of autophagy-controlling lipids. Our study has identified three lipids, 5OE, HSA, and SA, with potent anti-autophagic activity when applied to cultured dopaminergic cells at physiological concentrations found in human serum. In addition, this work has uncovered FABP5 as a regulator of autophagy, likely through the activity of lipid cargo.

5OE and AA are both well-established signaling lipids that were significant hits in our FABP5 interactome screen. AA also serves as the precursor for a diverse set of bioactive lipid signaling molecules making it of great interest in human physiology and disease [30, 31]. Nevertheless, since we do not observe a direct effect on autophagy in our system, we did not explore AA biology further. We do note, however, that AA is an interactor of FABP5 and a precursor for 5OE, which provides additional evidence for a physiological FABP5-5OE interaction. 5OE can be produced and released locally or as a precursor metabolite (5-HETE) released and metabolized by the target cell into 5OE (via transcellular biosynthesis) [32, 33]. 5OE has been well-studied for its role as an inflammatory cytokine that can affect a wide range of leukocytes [34]. However, 5OE is reported to exhibit the greatest potency on eosinophils as both a chemotactic agent and a stimulant, suggesting an anti-parasitic or allergic response role [32]. Interestingly, previous work has uncovered another allergy response pathway expressed by dopaminergic neurons that sensitizes these cells to death [35]. This raises the question of whether dopaminergic neurons are susceptible to stress-induced by allergic response signaling. A possible explanation for the heightened expression of FABP5 in dopaminergic neurons of the substantia nigra **(Fig 1)** is to sequester 5OE due to enhanced sensitivity toward this mode of allergic response signaling.

Our data support a direct role for 5OE upon dopaminergic neurons, which is consistent with reports of other proinflammatory cytokines (e.g., IL-6, TNF-alpha) [36]. While there are reports of an oxoeicosanoid receptor 1 (OXER1) [37] that can interact with 5OE as well as other eicosanoids, leukotrienes, and 5OE metabolites [38], it is likely, not responsible for autophagy inhibition by 5OE that we observe. This is because OXER1 is a plasma membrane-bound G-protein-coupled receptor, while our isolated FABP5-5OE complexes reside within the cytoplasm (mild, detergent-free immunoprecipitation). We have also observed that FABP5 suppression by shRNA inhibits autophagy, indicating a cytoplasmic role for 5OE.

HSA and SA are long-chain fatty acids with an 18-carbon backbone that were also identified by our FABP5 lipidomic interactome screen. SA is a lipid commonly found at high levels in a large number of food products, including grains, milk, meat, and dairy. Thus, all individuals likely have some exposure to this compound. There are no reports of direct toxicity from SA. However, mounting evidence implicates this dietary lipid as a potential regulator of cell cycle and tumorigenesis, albeit with conflicting reports [39]. Metabolic derangement has also been noted in cells and rodents following SA exposure [40]. Further study of SA is likely warranted given the ubiquitous presence of this lipid in food products and the newly found association with autophagy inhibition reported here.

The addition of a single hydroxyl group to SA at the 12th carbon results in the creation of HSA. While HSA is not a natural food compound, it is found in considerable amounts in a large number of consumer products and can be produced by microbiota from oleic acid and could provide a natural source of unknown dosage in animals [41]. The synthetic addition of the hydroxyl group to SA provides favorable handling characteristics (waxiness), leading to the wide adoption of HSA in cosmetic products (e.g., shampoo, underarm deodorants, lipsticks, and more). Similar to SA, the study of HSA has suggested its potential involvement in regulating tumorigenesis [42]. In line with this observation, we found a strong association with cell cycle alteration and oncogenic pathways in our RNA Seq analysis of HSA-treated cells. Interestingly, both SA and HSA exhibited strong associations with PPAR signaling pathways for KEGG and Reactome annotated pathways in our analysis. A growing body of evidence suggests that FABP5 participates in nuclear hormone receptor activity, which includes PPARδ [43]. Many of the molecular details of this interaction conferred by lipid-FABP5-PPARδ complexes have been previously described [17]. The binding of lipids to FABP5 results in cytoplasmic to nuclear translocation of this complex, which in-turn activates PPARδ. It is also reported that PPARδ is a potent regulator of autophagy [44]. Therefore, HSA and SA might regulate autophagy by directing FABP5 translocation and subsequent PPARδ regulation. In addition, a metabolite of AA, anandamide, has been shown to be a substrate of PPARδ through direct interaction with FABP5 which further links this pathway to lipid signaling [34]. Taken together, this suggests that the PPARδ signaling pathway is of interest for further study as a potential mechanism for autophagy inhibition by HSA and SA.

In summary, we report that FABP5 regulates autophagy in dopaminergic cells. Our subsequent lipidomic interactome screen identified five lipid FABP5 interactors. Of these interactors, 5OE, HSA and SA were confirmed to inhibit autophagy in dopaminergic neuron-like cells. RNA Seq analysis for 5OE yielded few clues to the potential mechanism of action due to low differential transcript expression versus control. Conversely, transcriptomic assessment for both HSA and SA indicates a strong association with altered PPAR signaling which provides an intriguing target for further investigation into HSA/SA-FABP5-mediated autophagy inhibition. Overall, these results indicate that the identification of lipids that modulate autophagy could be a fruitful avenue for developing novel therapeutics.

## Supporting information

S1 Fig Confirmation of FABP5 autophagy regulation using a second shRNA construct. **(A)** RA-differentiated SH-SY5Y cells were transduced with lentivirus containing scrambled (Cntrl) or a second FABP5 shRNA construct distinct from the shRNA shown in Fig 2. LC3B-II expression evaluated by western blot and **(B)** autophagic flux determined by densitometry of relative abundance of LC3B-II normalized to β-Actin. **(C)** Representative images for mCherry-GFP-LC3B that was transduced by lentivirus into retinoic acid (RA)-differentiated control or FABP5 knockdown (shRNA #2) as described in **A** is shown and then autophagosome (yellow) and lysosomes (red) quantified. **(D)** Graphical depiction of red and yellow puncta is presented. 12 cell images were taken per condition per biological replicate (3 biological replicates were performed). *p<0.05, error bars = SEM. **(E)** Representative images of SH-SY5Y cells stably expressing scrambled and two distinct FABP5 shRNA constructs in undifferentiated and RA-differentiated states. Scale bar = 25 μm.
(PDF)

S2 Fig RNA Seq volcano plots. Volcano plots for **(A)** 4 μM 5OE, **(B)** 400 μM HSA, **(C)** 400 μM SA and **(D)** 400 μM PA are shown (red dots = up-regulated transcripts, green dots = down-regulated transcripts).
(PDF)
