# Supplementary material for: FABP5-binding lipids regulate autophagy in differentiated SH-SY5Y cells
Source: PLoS One. 2024 Jun 20;19(6):e0300168. doi: 10.1371/journal.pone.0300168 (PMC11189175; doi:10.1371/journal.pone.0300168)
Supplement: S1 Fig — (A) RA-differentiated SH-SY5Y cells were transduced with lentivirus containing scrambled (Cntrl) or a second FABP5 shRNA construct distinct from the shRNA shown in Fig 2. LC3B-II expression evaluated by western blot and (B) autophagic flux determined by densitometry of relative abundance of LC3B-II normalized to β-Actin. (C) Representative images for mCherry-GFP-LC3B that was transduced by lentivirus into retinoic acid (RA)-differentiated control or FABP5 knockdown (shRNA #2) as described in A is shown and then autophagosome (yellow) and lysosomes (red) quantified. (D) Graphical depiction of red and yellow puncta is presented. 12 cell images were taken per condition per biological replicate (3 biological replicates were performed). *p<0.05, error bars = SEM. (E) Representative images of SH-SY5Y cells stably expressing scrambled and two distinct FABP5 shRNA constructs in undifferentiated and RA-differentiated states. Scale bar = 25 μm. (PDF) [file pone.0300168.s001.pdf]

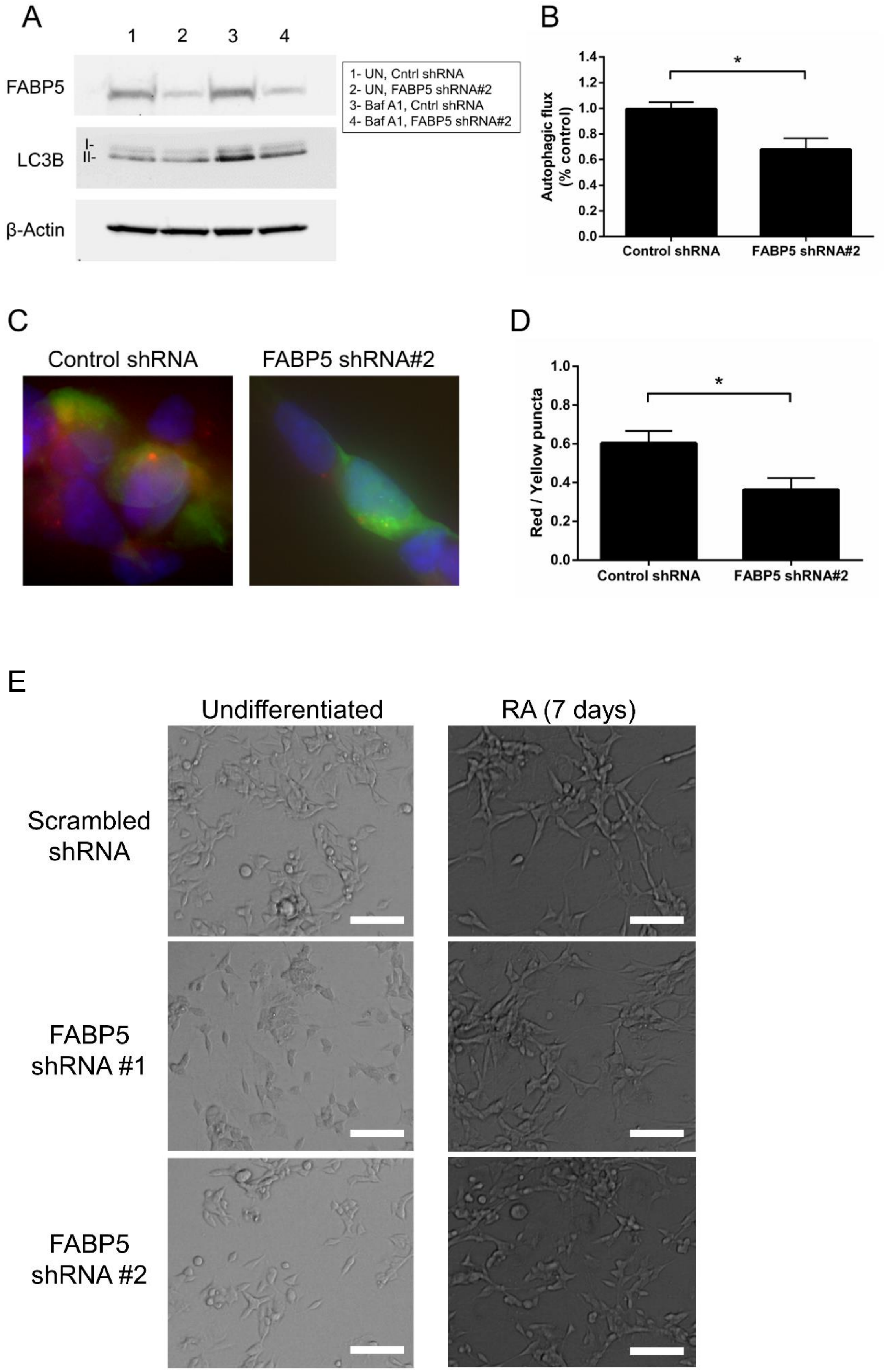
A
1
2
3
4
FABP5
LC3B
I-
II-
β-Actin
1- UN, Cntrl shRNA
2- UN, FABP5 shRNA#2
3- Baf A1, Cntrl shRNA
4- Baf A1, FABP5 shRNA#2
B
Autophagic flux (% control)
1.4
1.2
1.0
0.8
0.6
0.4
0.2
0.0
*
Control shRNA
FABP5 shRNA#2
C
Control shRNA
FABP5 shRNA#2
D
Red / Yellow puncta
1.0
0.8
0.6
0.4
0.2
0.0
*
Control shRNA
FABP5 shRNA#2
E
Undifferentiated
RA (7 days)
Scrambled shRNA
FABP5 shRNA #1
FABP5 shRNA #2

Figure S1. Confirmation of FABP5 autophagy regulation using a second shRNA construct. (A) RA-differentiated SH-SY5Y cells were transduced with lentivirus containing scrambled (Cntrl) or a second FABP5 shRNA construct distinct from the shRNA shown in Fig 2. LC3B-II expression evaluated by western blot and **(B)** autophagic flux determined by densitometry of relative abundance of LC3B-II normalized to β-Actin. **(C)** Representative images for mCherry-GFP-LC3B that was transduced by lentivirus into retinoic acid (RA)-differentiated control or FABP5 knockdown (shRNA #2) as described in **A** is shown and then autophagosome (yellow) and lysosomes (red) quantified. **(D)** Graphical depiction of red and yellow puncta is presented. 12 cell images were taken per condition per biological replicate (3 biological replicates were performed). *$p<0.05$, error bars = SEM. **(E)** Representative images of SH-SY5Y cells stably expressing scrambled and two distinct FABP5 shRNA constructs in undifferentiated and RA-differentiated states. Scale bar = 25 μm.
