# Supplementary material for: FABP5-binding lipids regulate autophagy in differentiated SH-SY5Y cells
Source: PLoS One. 2024 Jun 20;19(6):e0300168. doi: 10.1371/journal.pone.0300168 (PMC11189175; doi:10.1371/journal.pone.0300168)
Supplement: S2 Fig — Volcano plots for (A) 4 μM 5OE, (B) 400 μM HSA, (C) 400 μM SA and (D) 400 μM PA are shown (red dots = up-regulated transcripts, green dots = down-regulated transcripts). (PDF) [file pone.0300168.s002.pdf]

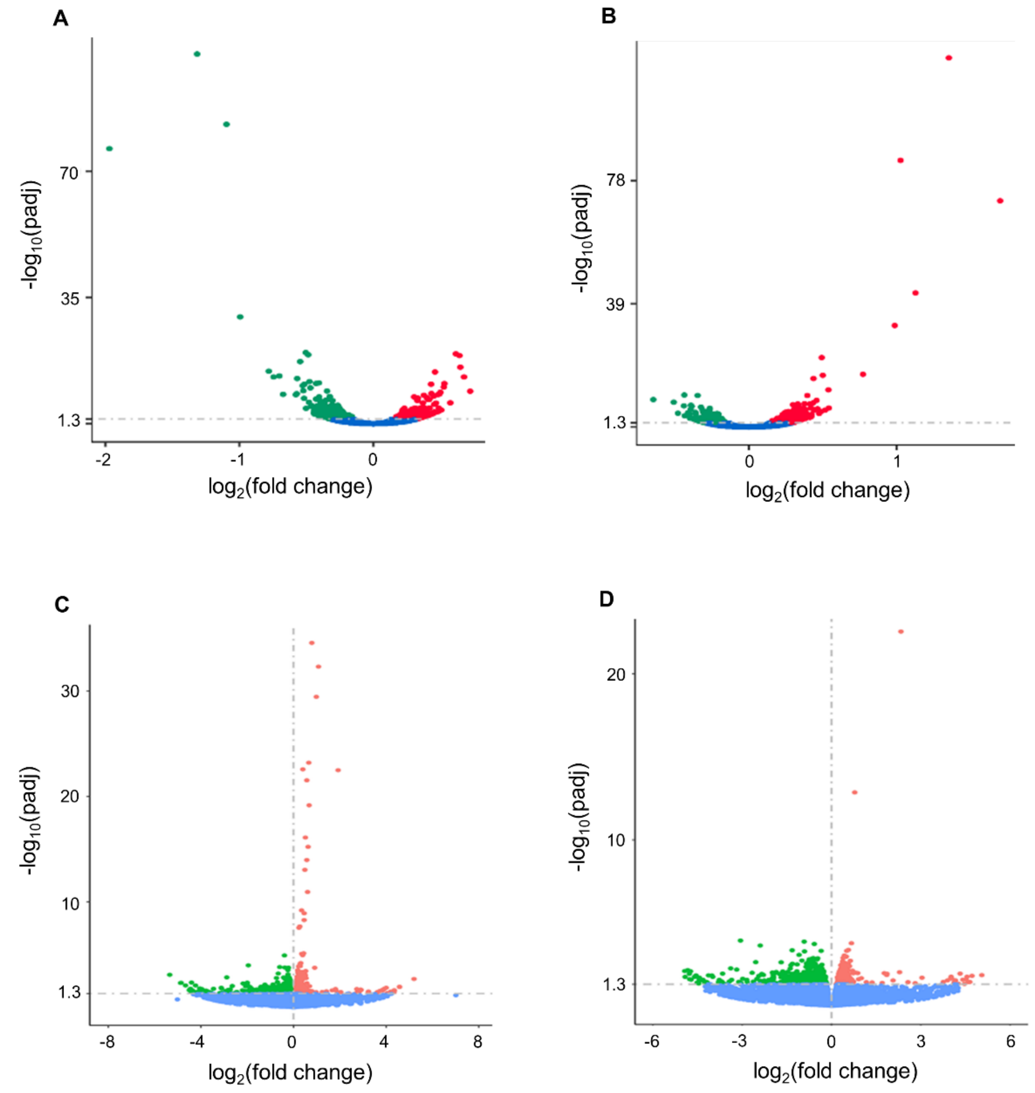

Figure S2. RNA Seq volcano plots. Volcano plots for **(A)** 4 µM 5OE, **(B)** 400 µM HSA, **(C)** 400 µM SA and **(D)** 400 µM PA are shown (red dots = up-regulated transcripts, green dots = down-regulated transcripts).
